# Long-term outcomes after extracorporeal membrane oxygenation in patients with dialysis-requiring acute kidney injury: A cohort study

**DOI:** 10.1371/journal.pone.0212352

**Published:** 2019-03-13

**Authors:** Shao-Wei Chen, Yueh-An Lu, Cheng-Chia Lee, An-Hsun Chou, Victor Chien-Chia Wu, Su-Wei Chang, Pei-Chun Fan, Ya-Chung Tian, Feng-Chun Tsai, Chih-Hsiang Chang

**Affiliations:** 1 Department of Cardiothoracic and Vascular Surgery, Chang Gung Memorial Hospital, Linkou Medical Center, Taoyuan, Taiwan; 2 Graduate Institute of Clinical Medical Science, College of Medicine, Chang Gung University, Taoyuan, Taiwan; 3 Kidney Research Center, Department of Nephrology, Change Gung Memorial Hospital, Linkou branch, Taoyuan, Taiwan; 4 Department of Anesthesiology, Chang Gung Memorial Hospital, Linkou Medical Center, Chang Gung University, Taoyuan City, Taiwan; 5 Department of Cardiology, Chang Gung Memorial Hospital, Keelung Branch and Linkou Medical Center, Taoyuan City, Taiwan; 6 Clinical Informatics and Medical Statistics Research Center, College of Medicine, Chang Gung University, Taoyuan, Taiwan; 7 Division of Allergy, Asthma, and Rheumatology, Department of Pediatrics, Chang Gung Memorial Hospital, Taoyuan, Taiwan; University of Sao Paulo Medical School, BRAZIL

## Abstract

**Background:**

Acute kidney injury (AKI) is a common complication of extracorporeal membrane oxygenation (ECMO) treatment. The aim of this study was to elucidate the long-term outcomes of adult patients with AKI who receive ECMO.

**Materials and methods:**

The study analyzed encrypted datasets from Taiwan’s National Health Insurance Research Database. The data of 3251 patients who received first-time ECMO treatment between January 1, 2003, and December 31, 2013, were analyzed. Characteristics and outcomes were compared between patients who required dialysis for AKI (D-AKI) and those who did not in order to evaluate the impact of D-AKI on long-term mortality and major adverse kidney events.

**Results:**

Of the 3251 patients, 54.1% had D-AKI. Compared with the patients without D-AKI, those with D-AKI had higher rates of all-cause mortality (52.3% vs. 33.3%; adjusted hazard ratio [aHR] 1.82, 95% confidence interval [CI] 1.53–2.17), chronic kidney disease (13.7% vs. 8.1%; adjusted subdistribution HR [aSHR] 1.66, 95% CI 1.16–2.38), and end-stage renal disease (5.2% vs. 0.5%; aSHR 14.28, 95% CI 4.67–43.62). The long-term mortality of patients who survived more than 90 days after discharge was 22.0% (153/695), 32.3% (91/282), and 50.0% (10/20) in the patients without D-AKI, with recovery D-AKI, and with nonrecovery D-AKI who required long-term dialysis, respectively, demonstrating a significant trend (*P*for trend <0.001).

**Conclusion:**

AKI is associated with an increased risk of long-term mortality and major adverse kidney events in adult patients who receive ECMO.

## Introduction

Extracorporeal membrane oxygenation (ECMO) has become a crucial technique for circulatory and respiratory support in intensive care over the past 2 decades. In adult patients, ECMO is an efficacious tool for bridging to organ recovery or transplantation when conventional management fails in cases of cardiogenic shock, respiratory failure, trauma of the respiratory system, and extreme hypothermia.[1–4] The application of ECMO is limited by its complications, including bleeding, stroke, infection, fasciotomy, amputation, massive blood transfusion, and acute kidney injury (AKI). Of these ECMO-related complications, AKI is the one most significantly associated with in-hospital mortality.[5]

AKI is a common complication of ECMO treatment. The reported incidence of new-onset AKI after ECMO in adults is 70.3%–84.4%, and approximately 60% of patients who receive ECMO require renal replacement therapy (RRT).[6–9] The cause of AKI in these patients is often multifactorial, involving both patient- and ECMO-related factors.[10] The severity of AKI is a strong predictor of adverse outcomes, and mortality is significantly higher in patients with AKI who require RRT.[11–13] Patients with AKI also exhibit long-term risk of developing chronic kidney disease (CKD), end-stage renal disease (ESRD), and death.[14, 15] A composite of renal outcomes, named major adverse kidney events (MAKE), has been endorsed by the National Institute of Diabetes and Digestive and Kidney Diseases clinical trials workgroup and used in recent studies.[16–19]

Evidence suggests that AKI influences patient outcomes after ECMO treatment. However, the long-term outcomes in survivors after catastrophic AKI and ECMO are not well described in the literature. Because ECMO treatment is associated with high mortality and morbidity, conducting a large-scale analysis of long-term ECMO outcomes in a single-center setting is difficult. Thus, this study used a nationwide database to comprehensively evaluate post-ECMO treatment events and the impact of AKI on patients who receive ECMO. The aim of this investigation was to elucidate the incidence, risk factors, hospitalization, and long-term outcomes of adult patients with ECMO who have advanced AKI.

## Materials and methods

### Data source

This study was performed by analyzing the National Health Insurance Research Database (NHIRD) of Taiwan. The NHIRD is derived from data collected as part of the National Health Insurance (NHI) program, which has provided compulsory universal health insurance for approximately 99.6% of Taiwan’s population since 1995. It contains comprehensive health care records that are released for medical research in the form of encrypted datasets. The NHI started providing reimbursements for ECMO therapy in December 2002. Researchers are able to elucidate the applications and patient outcomes of ECMO by analyzing datasets from the NHIRD.[20] The Institutional Review Board of Chang Gung Memorial Hospital approved this study and waived the need for individual informed consent.

### Study population

**Fig 1:**
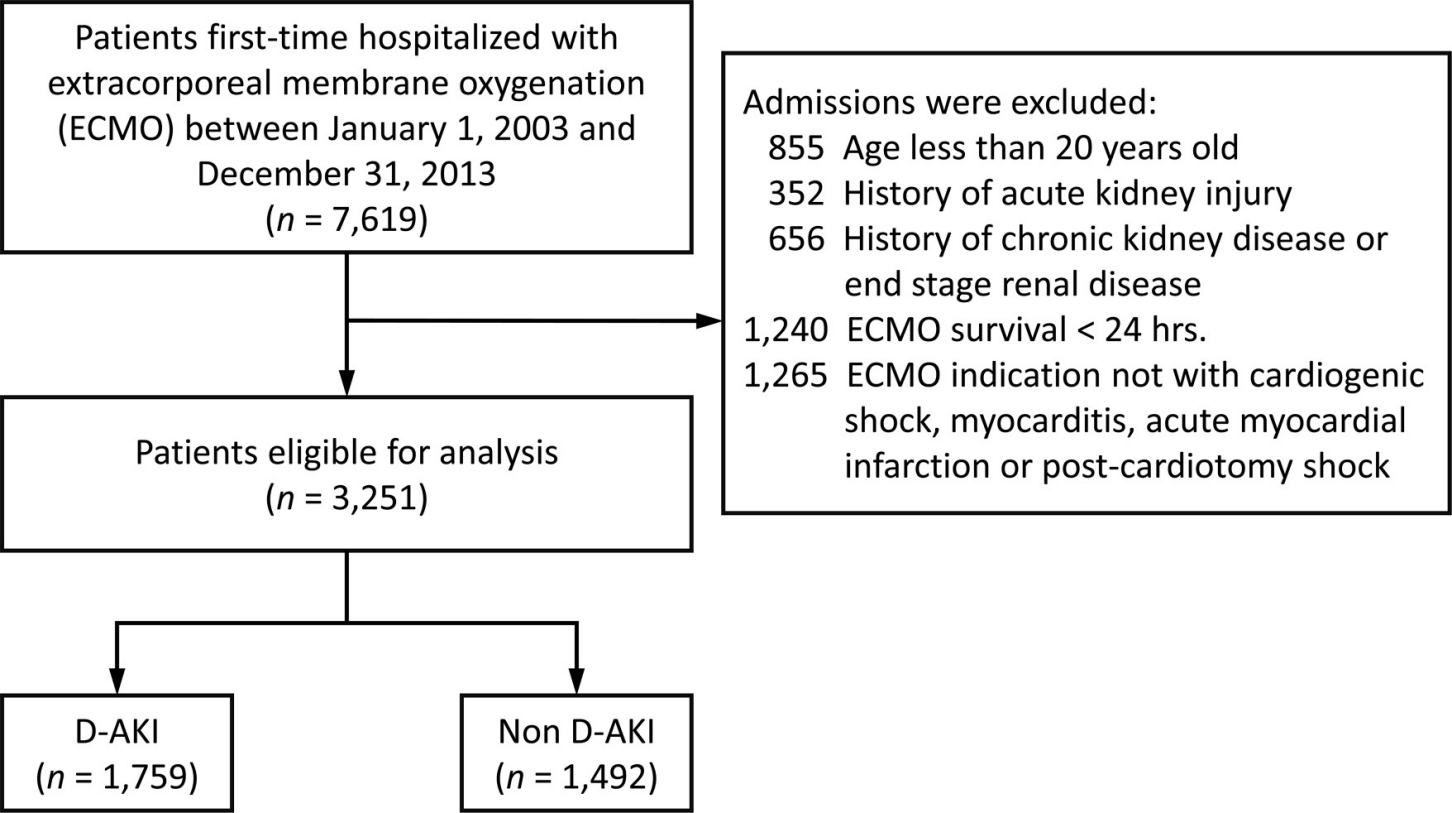
Patient’s inclusion criteria.

A flow chart of the patient inclusion process is shown in Fig 1. From the NHIRD, we retrieved the data of 7619 inpatients who received first-time ECMO treatment between January 1, 2003, and December 31, 2013. The hospitalization for first-time ECMO treatment was defined as the index admission. Because the study was aimed at investigating the long-term outcomes of new-onset dialysis-requiring acute kidney injury in adult patients who received ECMO, we excluded patients who had (1) an age less than 20 years (n = 855), (2) a history of AKI (n = 352), (3) a history of CKD or ESRD (n = 656), or (4) ECMO survival of less than 24 hours (n = 1240).

The datasets that obtained from NHIRD were unable to distinguish the veno-venous (VV) mode of ECMO from the veno-arterial (VA) configuration directly. VA mode ECMO is commonly used in patients with heart failure (such as cardiogenic shock, myocarditis, acute myocardial infraction and post-cardiotomy shock) and VV mode ECMO is usually applied in patients with respiratory failure (such as acute respiratory distress syndrome). [4, 21, 22] This study used ECMO indication as proxy variables for VA and VV modes and focused on those with VA mode ECMO. Patients who’s clinical indication of ECMO other than cardiogenic shock, myocarditis, acute myocardial infarction or post-cardiotomy shock (n = 1265) were excluded. A total of 3251 adult patients who received ECMO were eligible for the primary analysis. They were divided into a dialysis-requiring AKI (D-AKI) group and a non-D-AKI group depending on whether they newly started RRT during the index admission.

### Definitions of ECMO usage, D-AKI, comorbidities, and outcomes

The initiation and maintenance of the ECMO therapy is identified by *International Classification of Diseases*, *Ninth Revision*, *Clinical Modification* (*ICD-9-CM*) procedure code 39.65 and confirmed by ECMO-associated reimbursement codes in the NHIRD. We defined D-AKI as new-onset dialysis requirement during hospitalization, as identified by the reimbursement codes of RRT, namely continuous RRT, intermittent hemodialysis, and sustained low-efficiency daily dialysis. The demographic results were recorded from the NHIRD at the admission date of index admission. Comorbidities were defined using the *ICD-9-CM* diagnostic codes of medical records before the index admission (S1 Table).

Secondary outcomes were in-hospital complications during the index admission. The outcomes of primary interest in this study were long-term mortality and MAKE after discharge. Death was identified when an individual was withdrawn from the NHI system.[23] A MAKE was defined as a composite outcome of all-cause mortality, AKI, CKD, and ESRD.[18, 24] Incident AKI and CKD were identified using inpatient diagnoses after discharge of index admission. Patients were verified as having ESRD (requiring long-term dialysis) if they possessed a catastrophic illness certificate (CIC) for ESRD; this certification requires authentication by 2 nephrologists. We identified nonrecovery and recovery AKI according to whether a patient obtained a CIC for ESRD within the index admission (applied CIC for permanent dialysis during the hospital days) or within 3 months after discharge, respectively. The discharge date of index admission was assigned as the index date when analyzing patient’s long-term outcomes after discharge. Patients who survived in the index admission were followed from the index date to the date of event occurrence or December 31, 2013. The follow up year was the length from the index date to death or December 31, 2013 where the follow up duration of patients who died during the index admission was zero.

### Statistical analysis

Continuous data are expressed as the mean ± standard deviation (SD) and categorical data are expressed as the frequency and percentage (%) of each baseline characteristic. Differences between the D-AKI and non-D-AKI groups were compared using the chi-square and Student *t* tests for categorical and continuous variables, respectively. In-hospital outcomes were compared between the 2 groups by using logistic regression analysis for the categorical variables and linear regression analysis for the continuous variables. To determine the risk factors for D-AKI during the index admission, we performed multivariable logistic regression analysis that included the baseline characteristics as covariates and adopted a backward elimination procedure. We subsequently focused on patients who survived the index admission and compared the long-term outcomes of the 2 groups. We used Cox proportional hazard models to compare the risk of all-cause mortality and MAKE between the 2 groups. Patients who expired at discharge were excluded from the MAKE survival analysis. For AKI, CKD, and ESRD, we used subdistribution hazard models that considered death during follow-up as a competing risk [25] The aforementioned regression models, including the logistic, linear, Cox regression, and subdistribution hazard models adjusted for baseline characteristics. The survival rates in terms of MAKE and all-cause mortality were calculated and compared between the 2 groups by using log-rank test. The cumulative incidences of ESRD in the groups were determined and compared using a competing risk survival model. Finally, long-term mortality rates stratified by renal function were compared using a log-rank trend test. A two-sided *P* value of <0.05 was considered statistically significant. Data analyses were conducted using SAS software version 9.4 (SAS Institute, Cary, NC).

### Validation

To verify the accuracy of the primary variables and ensure the internal validity of this study, an internal validation was performed. The validation was conducted using a chart review of consecutive patients who received first-time ECMO treatment between January 2011 and December 2012 in a tertiary medical center (Chang Gung Memorial Hospital, Taoyuan, Taiwan). The patients in the chart review were linked to those in the NHIRD according to birth date, sex, admission date, and discharge date. We validated the 2 variables of primary interest. First, we validated the definition of D-AKI, which was the main exposure of this study. Second, because death is our primary outcome, we chose to validate in-hospital mortality. After linking the 2 sources, we determined the sensitivity, specificity, positive predictive value (PPV), and negative predictive value of D-AKI and in-hospital mortality between the chart review and NHIRD patients.[26]

### Long-term outcomes of patients with all indications of ECMO

The primary analysis of the study focused on patients with VA mode ECMO. To further understand the clinical application of our results, we performed a sensitivity analysis in patients with all indications of ECMO. In addition to patients with cardiogenic shock, myocarditis, acute myocardial infraction and post-cardiotomy shock, the sensitivity analysis involved patients with ECMO indication of respiratory failure, trauma and other. The flow chart of patient inclusion is shown in S1 Fig.

## Results

**Fig 2:**
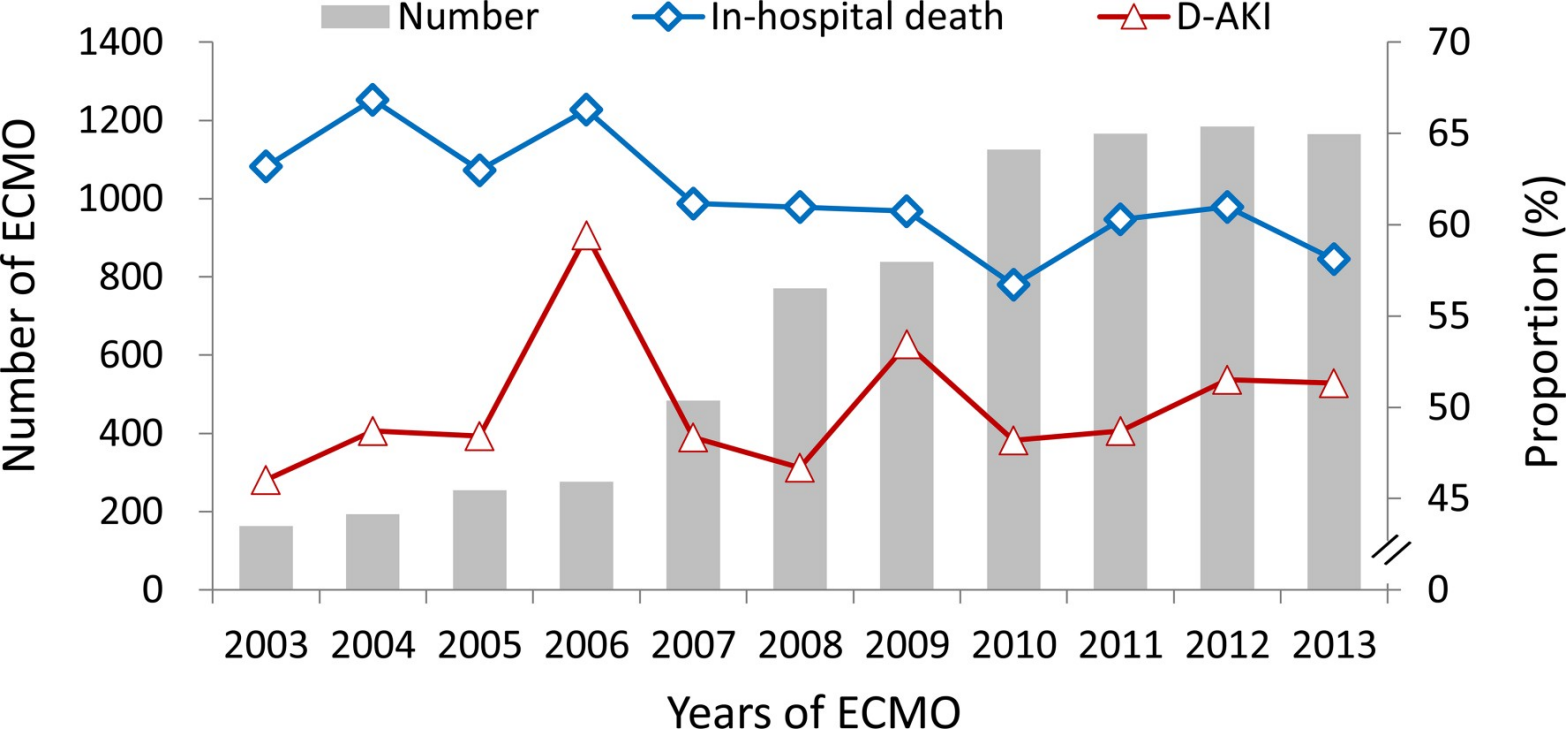
The number of ECMO, proportion of D-AKI (P trend = 0.415) and proportion of in-hospital mortality (P trend = 0.007) between 2003 and 2013.

In this study, 3251 patients who received first-time ECMO treatment between January 1, 2003, and December 31, 2013, were identified as being eligible for analysis. Of these patients, 54.1% (n = 1759) developed D-AKI during the index admission (Fig 1). The number of all patients with ECMO (n = 7619), their proportion of D-AKI, and the proportion of in-hospital deaths during the study period are summarized in Fig 2. Although the rate of in-hospital mortality decreased (*P* of trend test = 0.007), the incidence of D-AKI did not change significantly throughout the 11 years (*P* of trend test = 0.415).

Compared with the non-D-AKI group, the D-AKI group was older; had higher rates of ECMO indication as postcardiotomy shock, history of heart failure, prior myocardial infraction, prior stroke, and liver cirrhosis; exhibited a higher average Charlson score; was more likely to be admitted in a medical center than in a district / regional hospital; and had a shorter follow-up duration (Table 1). In addition, the patients with D-AKI had significantly higher rates of in-hospital mortality, sepsis, fasciotomy or amputation, respiratory failure, intra-aortic balloon pump usage and massive blood transfusion (pack red blood cell > 10 units), as well as more transfusion of blood preparations, longer ECMO and ventilator support duration, longer intensive care unit (ICU) stay, shorter hospital stay, and more inpatient medical expenditure (Table 2). The following risk factors were significantly associated with the development of D-AKI: older age, medical center hospitalization, heart failure, liver cirrhosis, and ECMO indication as postcardiotomy shock (Table 3).

**Table 1:** Demographic and clinical characteristics of the study population.

Variable		Total (*n* = 3,251)	D-AKI (*n* = 1,759)	Non D-AKI (*n* = 1,492)	*P*
Age (years)		57.5±15.8	58.4±15.8	56.4±15.7	<0.001
Age group					0.007
	≤ 40 yrs.	493 (15.2)	249 (14.2)	244 (16.4)	
	41–50 yrs.	490 (15.1)	255 (14.5)	235 (15.8)	
	51–60 yrs.	798 (24.5)	409 (23.3)	389 (26.1)	
	61–70 yrs.	665 (20.5)	367 (20.9)	298 (20.0)	
	71–80 yrs.	583 (17.9)	350 (19.9)	233 (15.6)	
	> 80 yrs.	222 (6.8)	129 (7.3)	93 (6.2)	
Gender					0.955
	Male	2,320 (71.4)	1,256 (71.4)	1,064 (71.3)	
	Female	931 (28.6)	503 (28.6)	428 (28.7)	
ECMO indication					<0.001
	CV (Cardiogenic shock, myocarditis or AMI)	1,209 (37.2)	597 (33.9)	612 (41.0)	
	Post-cardiotomy shock	2,042 (62.8)	1,162 (66.1)	880 (59.0)	
Comorbid conditions					
	Diabetes mellitus	844 (26.0)	474 (26.9)	370 (24.8)	0.164
	Hypertension	1,214 (37.3)	652 (37.1)	562 (37.7)	0.724
	Heart failure	672 (20.7)	408 (23.2)	264 (17.7)	<0.001
	Coronary artery disease	2,071 (63.7)	1,127 (64.1)	944 (63.3)	0.637
	Prior myocardial infarction	437 (13.4)	264 (15.0)	173 (11.6)	0.004
	Atrial fibrillation	358 (11.0)	203 (11.5)	155 (10.4)	0.296
	Peripheral arterial disease	111 (3.4)	63 (3.6)	48 (3.2)	0.569
	Prior stroke	325 (10.0)	199 (11.3)	126 (8.4)	0.007
	Coagulopathy	111 (3.4)	63 (3.6)	48 (3.2)	0.569
	Chronic obstructive pulmonary disease	180 (5.5)	109 (6.2)	71 (4.8)	0.074
	Liver cirrhosis	55 (1.7)	40 (2.3)	15 (1.0)	0.005
	Malignancy	135 (4.2)	72 (4.1)	63 (4.2)	0.854
	Charlson’s score	2.2±1.8	2.4±1.9	2.0±1.7	<0.001
Study year					0.044
	2003–2006	417 (12.8)	222 (12.6)	195 (13.1)	
	2007–2010	1,327 (40.8)	687 (39.1)	640 (42.9)	
	2011–2013	1,507 (46.4)	850 (48.3)	657 (44.0)	
Hospital level					<0.001
	Medical center	2,379 (73.2)	1,336 (76.0)	1,043 (69.9)	
	District / regional hospital	872 (26.8)	423 (24.0)	449 (30.1)	
Follow-up years		1.0±2.0	0.5±1.5	1.5±2.3	<0.001

D-AKI, dialysis-dependent acute kidney injury; ECMO, extracorporeal membrane oxygenation; CV, cardiovascular; AMI, acute myocardial infarction.

**Table 2:** In-hospital outcomes.

		Total (*n* = 3,251)	D-AKI (*n* = 1,759)	Non D-AKI (*n* = 1,492)	D-AKI *vs*. Non D-AKI	
Outcome					*B* / OR (95% CI)^1^	*P* value
**Categorical parameter**						
	**In-hospital mortality**	1,928 (59.3)	1,298 (73.8)	630 (42.2)	3.92 (3.36–4.57)	<0.001
	**New onset stroke**	211 (6.5)	107 (6.1)	104 (7.0)	0.77 (0.58–1.04)	0.087
	**New onset ischemic** **stroke**	147 (4.5)	77 (4.4)	70 (4.7)	0.85 (0.60–1.20)	0.342
	**New onset** **hemorrhagic stroke**	71 (2.2)	32 (1.8)	39 (2.6)	0.64 (0.39–1.04)	0.071
	**Sepsis**	487 (15.0)	323 (18.4)	164 (11.0)	1.85 (1.50–2.27)	<0.001
	**Fasciotomy or** **amputation**	55 (1.7)	38 (2.2)	17 (1.1)	1.85 (1.02–3.36)	0.043
	**Respiratory failure**	484 (14.9)	301 (17.1)	183 (12.3)	1.34 (1.09–1.64)	0.005
	**IABP**	1,879 (57.8)	1,037 (59.0)	842 (56.4)	1.18 (1.01–1.38)	0.039
	**Massive blood** **transfusion, PRBC >** **10 Units**	1,967 (60.5)	1,248 (70.9)	719 (48.2)	2.80 (2.37–3.31)	<0.001
**Continuous parameter**						
	**PRBC amount**	19.1±17.9	23.1±19.6	14.4±14.3	7.28 (6.17–8.39)	<0.001
	**FFP amount**	14.1±20.5	18.2±23.7	9.1±14.5	7.66 (6.35–8.97)	<0.001
	**Platelet amount**	11.8±20.0	14.6±22.6	8.6±15.6	5.99 (4.68–7.30)	<0.001
	**ECMO support** **duration (days)**	5.2±3.1	5.7±3.4	4.7±2.6	0.81 (0.60–1.02)	<0.001
	**Ventilator (days)**	14.4±16.2	16.0±17.9	12.5±13.7	2.72 (1.61–3.82)	<0.001
	**ICU duration (days)**	16.1±16.8	17.4±18.3	14.5±14.7	1.97 (0.83–3.10)	<0.001
	**Hospital stays (days)**	26.3±31.4	26.0±32.9	26.6±29.7	-2.55 (-4.67– -0.44)	0.018
	**Inpatient medical** **expenditure (NTD×10**^**4**^**)**	95.7±70.1	107.5±76.7	81.8±58.5	19.57 (15.17–23.97)	<0.001

^1^ Adjusted for variables listed in Table 1 except follow up years

D-AKI, dialysis-dependent acute kidney injury; *B*, regression coefficient; OR, odds ratio; CI, confidence interval; IABP, intra-aortic balloon pump; PRBC, packed red blood cells; FFP, fresh frozen plasma; ICU, intensive care unit; NTD, New Taiwan Dollar.

**Table 3:** Risk factor analysis of D-AKI.

Variable		Odds ratio	95% CI	*P* value
**Age (per 10 years)**		1.080	1.033–1.130	<0.001
**Indication (Ref: CV)**				
	**Post-cardiotomy shock**	1.297	1.121–1.502	<0.001
**Heart failure**		1.345	1.128–1.604	0.001
**Liver cirrhosis**		2.076	1.135–3.797	0.018
**Study year (Ref: 2003–2006)**				
	**2007–2010**	1.031	0.823–1.292	0.789
	**2011–2013**	1.277	1.021–1.598	0.032
**Medical center**		1.355	1.156–1.589	<0.001

D-AKI, dialysis-dependent acute kidney injury; CI, confidence interval; CV, cardiovascular.

**Fig 3:**
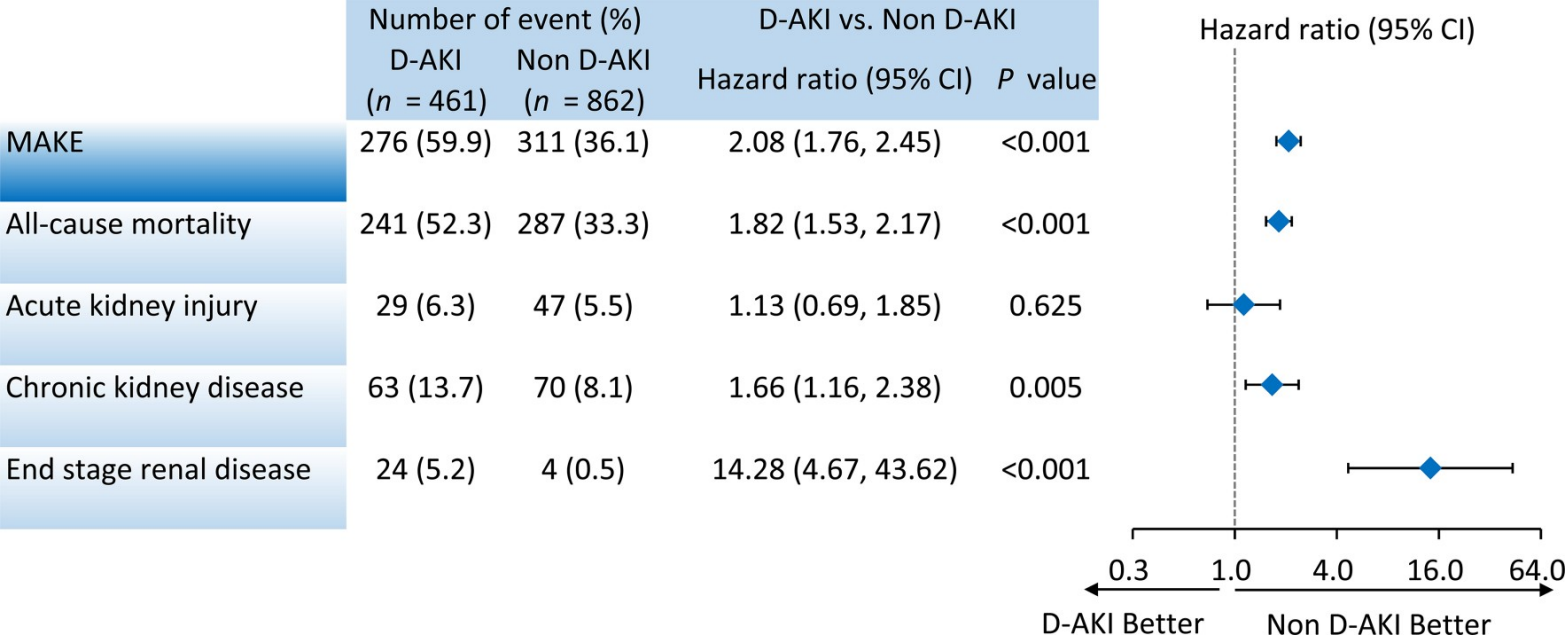
Primary result of major adverse kidney events (MAKE) and each MAKE component.

**Fig 4:**
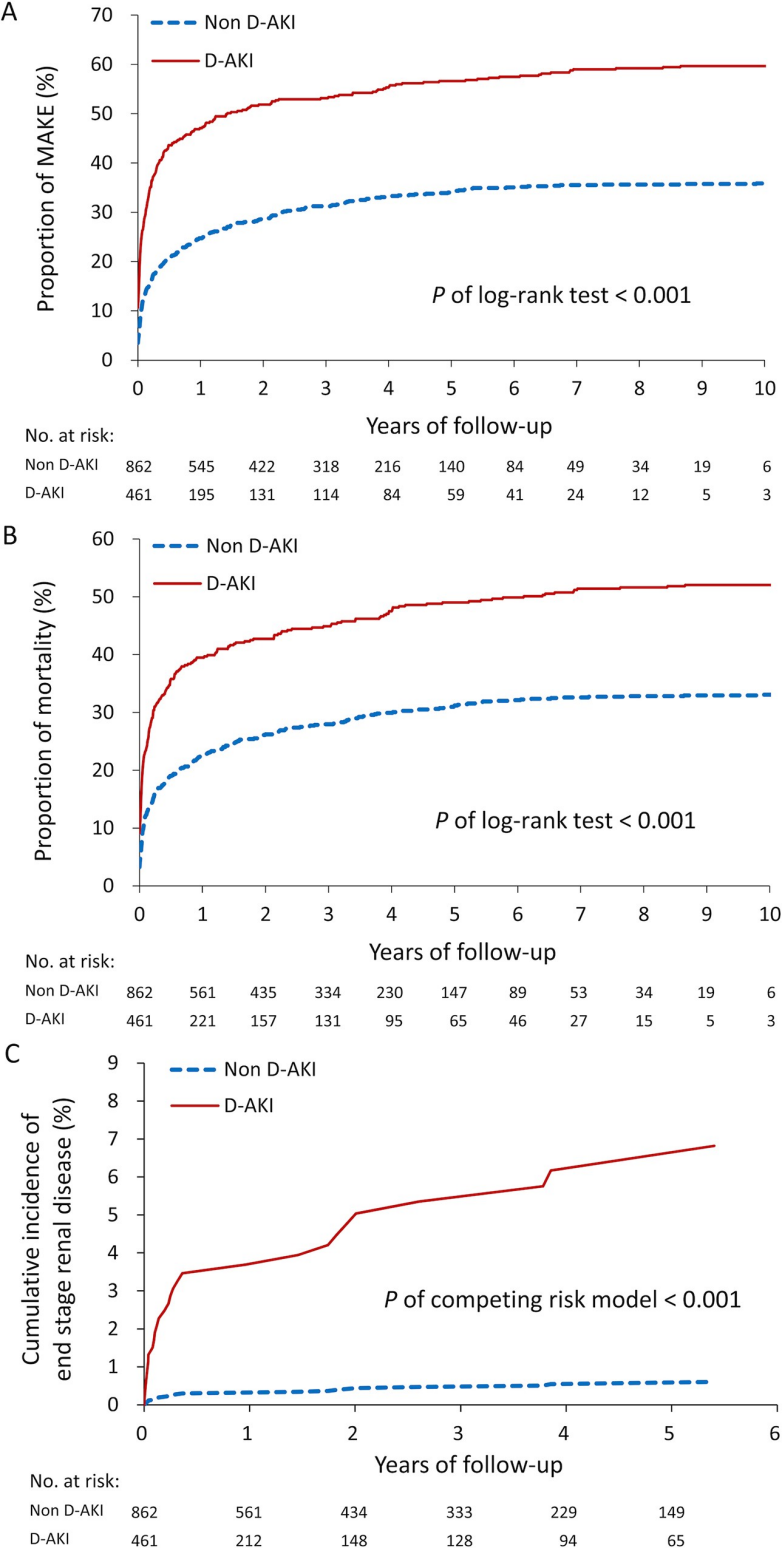
Proportion of major adverse kidney events (A) and all-cause mortality (B), cumulative incidence of end stage renal disease (C) during follow-up in the D-AKI and non D-AKI patients.

The long-term outcomes of those who survived during the index admission are presented in Fig 3. During the mean follow-up period of 2.4±2.5 years, the patients with D-AKI had a higher risk of MAKE than did the patients without D-AKI (59.9% vs. 36.1%; adjusted hazard ratio [aHR] 2.08, 95% confidence interval [CI] 1.76–2.45). In the advanced analysis of each MAKE component, the patients with D-AKI exhibited a higher all-cause mortality rate (52.3% vs. 33.3%; aHR 1.82, 95% CI 1.53–2.17) as well as higher cumulative incidences of new-onset CKD (13.7% vs. 8.1%; adjusted subdistribution HR [aSHR] 1.66, 95% CI 1.16–2.38) and ESRD (5.2% vs. 0.5%; aSHR 14.28, 95% CI 4.67–43.62). There was no significant difference in AKI (6.3% vs. 5.5%; aSHR 1.13, 95% CI 0.69–1.85). The Kaplan–Meier survival rate of MAKE and all-cause mortality as well as the cumulative incidence of ESRD during the follow-up period are depicted in Fig 4A, 4B, and 4C, respectively. The patients with D-AKI had significantly poorer prognostic outcomes.

**Fig 5:**
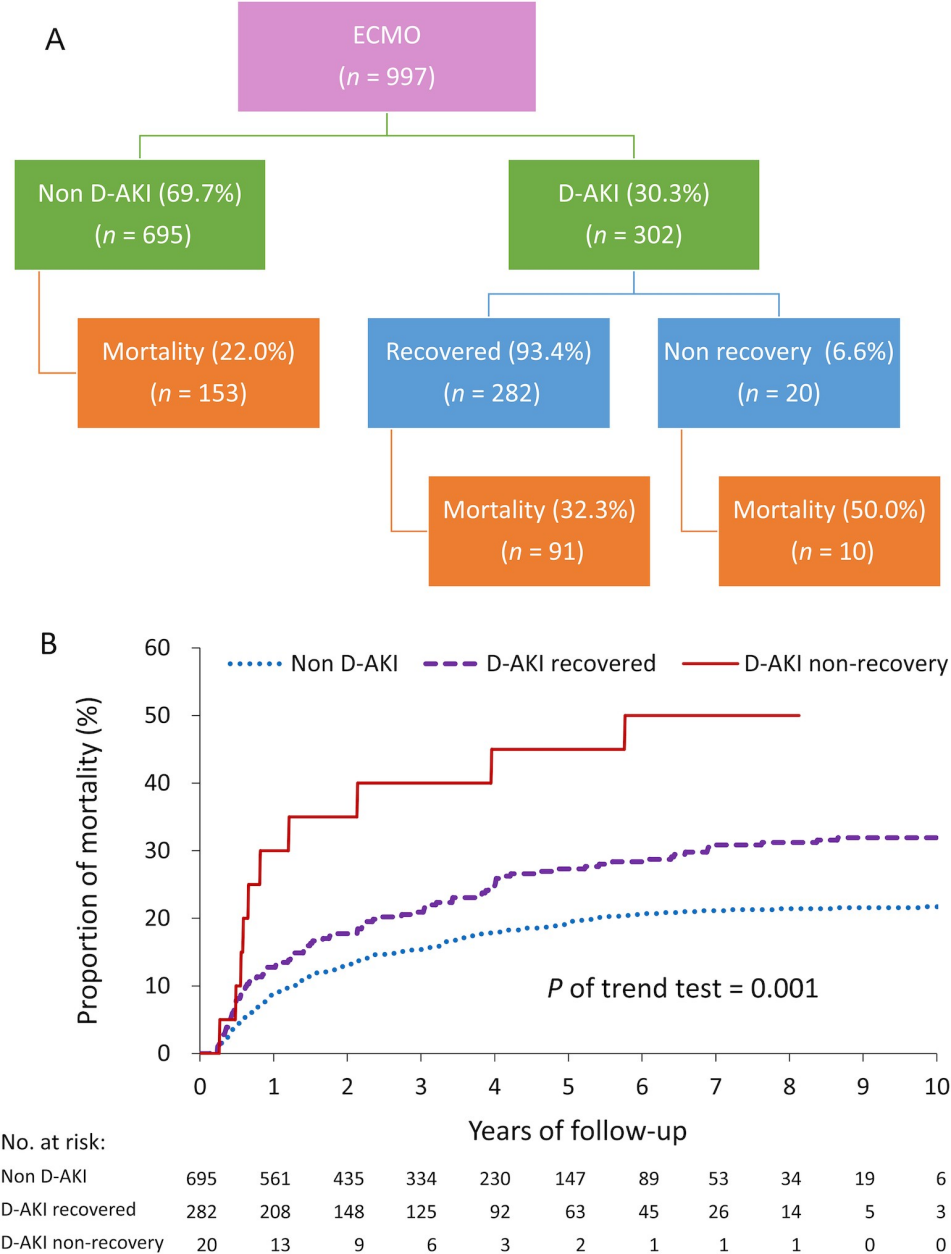
Patients who discharged from the index admission and were alive within 90 days after discharge. The mortality rate was shown by the stratification of renal function (A) along with a log-rank trend test (B).

For the patients who were discharged after the index admission and were alive within 90 days after discharge (n = 997), the mortality rate as stratified by renal function is presented in Fig 5A. The mortality rates were 22.0% (153/695), 32.3% (91/282), and 50.0% (10/20) for the patients without D-AKI, with recovery D-AKI, and with nonrecovery D-AKI who required long-term dialysis, respectively. Log-rank trend test revealed that the mortality rate during the follow-up period was significantly higher in the patients with D-AKI, particularly among those who had nonrecovery D-AKI after discharge (*P* of trend test = 0.001, Fig 5B).

For the internal validation chart review, we enrolled 151 patients in our hospital who received first-time ECMO between 2011 and 2012. Regarding the validation of D-AKI, 82 of the 85 patients who were identified as D-AKI in the NHIRD met the criteria for having D-AKI in our chart review. Fifty-eight of the 66 patients who were classified in the non-D-AKI group based on the NHIRD were confirmed in the chart review to have not experienced RRT during admission. Regarding the validation of in-hospital mortality, 80 of the 85 patients who were classified as nonsurvivors in the NHIRD database were confirmed to have expired in the chart review. Fifty-six of the 66 patients who were identified as survivors in the NHIRD were found to have indeed survived when discharged. The PPVs of D-AKI and in-hospital mortality in the NHIRD were 96.47% and 94.12%, respectively (S2 Fig).

The results of sensitivity analysis of patients with all indication of ECMO were showed in supplementary tables and figures (S2–S4 Tables, S3–S5 Figs). Patients with D-AKI had a higher risk of MAKE than did the patients without D-AKI (57.2% vs. 34.8%; aHR 1.98, 95% CI 1.72–2.29). In the advanced analysis of each MAKE component, the patients with D-AKI exhibited a higher all-cause mortality rate (49.9% vs. 31.8%; aHR 1.79, 95% CI 1.54–2.08) as well as higher cumulative incidences of new-onset CKD (12.8% vs. 7.9%; aHR 1.52, 95% CI 1.11–2.08) and ESRD (5.3% vs. 0.5%; aHR 9.55, 95% CI 3.99–22.85). There was no significant difference in AKI (6.2% vs. 4.9%; aHR 1.18, 95% CI 0.77–1.80). The mortality rates of patients who were discharged after the index admission and were alive within 90 days after discharge were 20.8%, 32.3%, and 42.3% for the patients without D-AKI, with recovery D-AKI, and with nonrecovery D-AKI who required long-term dialysis. Results of the sensitivity analysis were compatible with that of the primary analysis and disclosed the possibility of application of our results in patients with all indications of ECMO treatment.

## Discussion

This population-based cohort study analyzed the data of 3251 patients who received first-time ECMO treatment between 2003 and 2013, as extracted from a nationwide database. Our results provide evidence that patients who receive ECMO and have D-AKI face poorer long-term outcomes in terms of MAKE and survival compared with patients without D-AKI. Survival rates differed significantly when patients were stratified into non-D-AKI, recovery D-AKI, and non-recovery D-AKI groups after discharge.

In Taiwan, the number of ECMO cases started increasing in 2007 and plateaued in 2010, with approximately 1150 cases yearly. Although the in-hospital survival rate has increased, probably because of improvements in ECMO techniques and the quality of patient care, patients who receive the treatment still exhibit a high incidence of developing D-AKI. Of the 3251 patients examined in this study, only 997 of them (30.7%) were alive after 90 days after being discharged. These results demonstrate that ECMO with D-AKI constitutes a catastrophic condition that warrants careful inspection and outpatient care in order to increase the likelihood of survival.

D-AKI is associated with an in creased risk of mortality and complications in patients received ECMO treatment.[27] ECMO can lead to AKI through exposure to non-self membranes, nonpulsatile renal blood flow, blood shear stress, air and blood embolisms, hemolysis, and organ crosstalk of the heart, lung, and kidney.[10, 13] Nevertheless, it is generally believed that patient-related factors, including hypoperfusion, hypoxemia, systemic inflammation, and nephrotoxic drug use, playing the main role of developing AKI in critically ill patients who receive ECMO.[27–29] Thus, it is not particularly surprising that in this study, heart failure and liver cirrhosis are major risk factors for developing D-AKI in patients who receive ECMO.

It is not easy to determine the optimal time of dialysis initiation in critical ill patients with AKI. Decreased urine output and fluid overload are associated with prolonged ECMO duration and poor clinical outcomes.[9, 30] Renal support in ECMO patients with AKI maintains fluid balance as well as reduces inflammatory mediators and metabolic waste. A meta-analysis suggested that RRT reduces mortality rates in patients receiving ECMO.[28] Our results showed an increased long-term mortality in D-AKI group. Early initiation of RRT to manage the fluid accumulation aggressively in patients with ECMO might be beneficial for patient’s outcomes.[31] The optimal timing of RRT initiation in these patients needs further study.

Long-term renal outcomes after ECMO treatment are often neglected. To our knowledge, the current study is the first to have demonstrated an association between AKI and long-term MAKE in patients who receive ECMO. *Corte et al*. reported that in ICU patients with D-AKI, the proportions of complete renal recovery, incomplete renal recovery, and dialysis dependence were 48.4%, 32.6%, and 19.0%, respectively, after 1 year of follow-up.[32] AKI is an independent predictor of CKD, ESRD, and death, and its impact attenuates over the course of a long follow-up.[15, 33–36] Nephron loss in AKI may result in glomerular hypertrophy, hyperfiltration, and systemic hypertension thus lead to the development of CKD and ESRD.[37] In addition to MAKE, AKI has also been associated with coronary artery events, infections, upper gastrointestinal hemorrhages, delayed recovery of physical function, and an increased cost burden on the health care system in long-term follow-ups.[21, 38–41] Due to the high prevalence of AKI after ECMO treatment, long-term monitoring of kidney function and associated complications is warranted.

This study was limited by the heterogeneity of the study cohort. We were unable to distinguish the VV mode of ECMO from the VA configuration in the NHIRD, and ECMO settings such as cannula size, cannula insertion site, and centrifugal pump speed were unknown. For this limitation, we have made efforts to identify VA mode by using indication as a proxy variable, but misclassification might exist. [21, 22] Furthermore, we were unable to evaluate the initial renal function and status of renal recovery because of the lack of blood investigation results. The date of dialysis initiation was uncertain in NHIRD, so that we were unable to state that every patient started dialysis after ECMO commencement. Except for this, our study provided outcomes of patients with ECMO who developed D-AKI during the index admission in a large-scale population. Because this study used *ICD-9-CM* diagnostic codes, reimbursement codes, and withdrawal from the NHI system to define comorbidities, MAKE, D-AKI, and death, some misclassifications may have occurred. Thus, we applied an internal validation of D-AKI and death. A strength of this study is in the application of this internal validation, which revealed adequate PPVs for both D-AKI and death.

In conclusion, D-AKI was associated with long-term mortality and MAKE in adult patients who received ECMO. In the advanced analysis of each MAKE component, the patients with D-AKI had a higher all-cause mortality rate as well as higher cumulative incidences of new-onset CKD and ESRD; however, there was no significant difference in AKI. Aggressive renal support should be considered when fluid accumulation occurs. Long-term monitoring of kidney function and associated complications is warranted in patients who receive ECMO.

## Supporting information

S1 Fig Patient's inclusion criteria of the analysis of patients with all indications of ECMO. (TIF)

S2 Fig Validation of D-AKI (A) and in-hospital mortality (B).
(TIF)

S3 Fig Result of major adverse kidney events (MAKE) and each MAKE component of patients with all indications of ECMO. (TIF)

S4 Fig Proportion of major adverse kidney events (A) and all-cause mortality (B), cumulative incidence of end stage renal disease (C) during follow-up in the D-AKI and non D-AKI patients of patients with all indications of ECMO.
(JPG)

S5 Fig Patients who discharged from the index admission and were alive within 90 days after discharge. The mortality rate was shown by the stratification of renal function (A) along with a log-rank trend test (B) of patients with all indications of ECMO.
(JPG)

S1 Table ICD-9-CM code used for diagnosis in the current study. (DOCX)

S2 Table Demographic and clinical characteristics of patients with all indications of ECMO. (DOCX)

S3 Table In-hospital outcomes of patients with all indications of ECMO. (DOCX)

S4 Table Risk factor analysis of D-AKI of patients with all indications of ECMO. (DOCX)
