# Supplementary figures and images for: Long-term outcomes after extracorporeal membrane oxygenation in patients with dialysis-requiring acute kidney injury: A cohort study
Source: PLoS One. 2019 Mar 13;14(3):e0212352. doi: 10.1371/journal.pone.0212352 (PMC6415889; doi:10.1371/journal.pone.0212352)

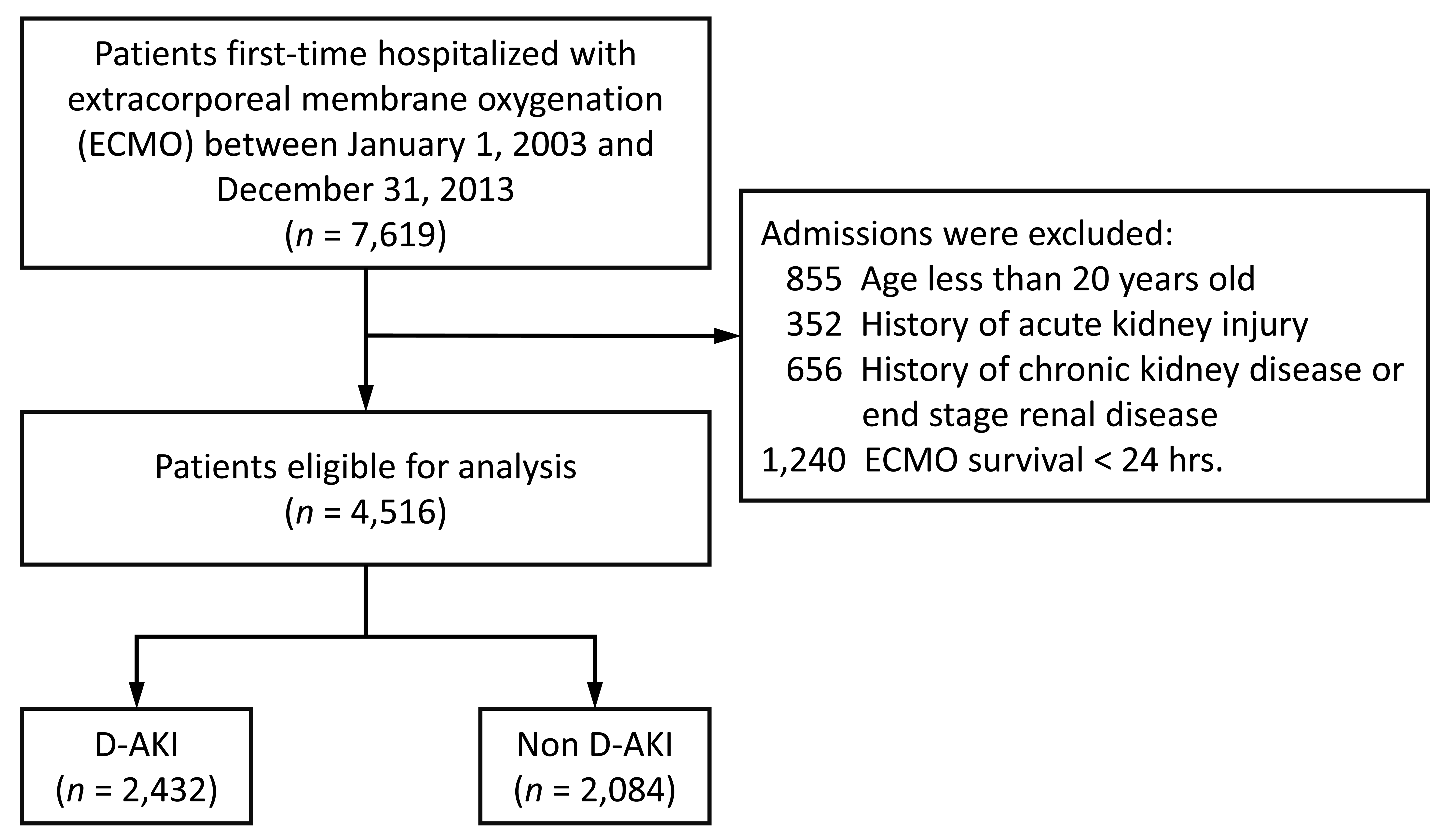

Supplement: S1 Fig — (TIF) [file pone.0212352.s001.tif]

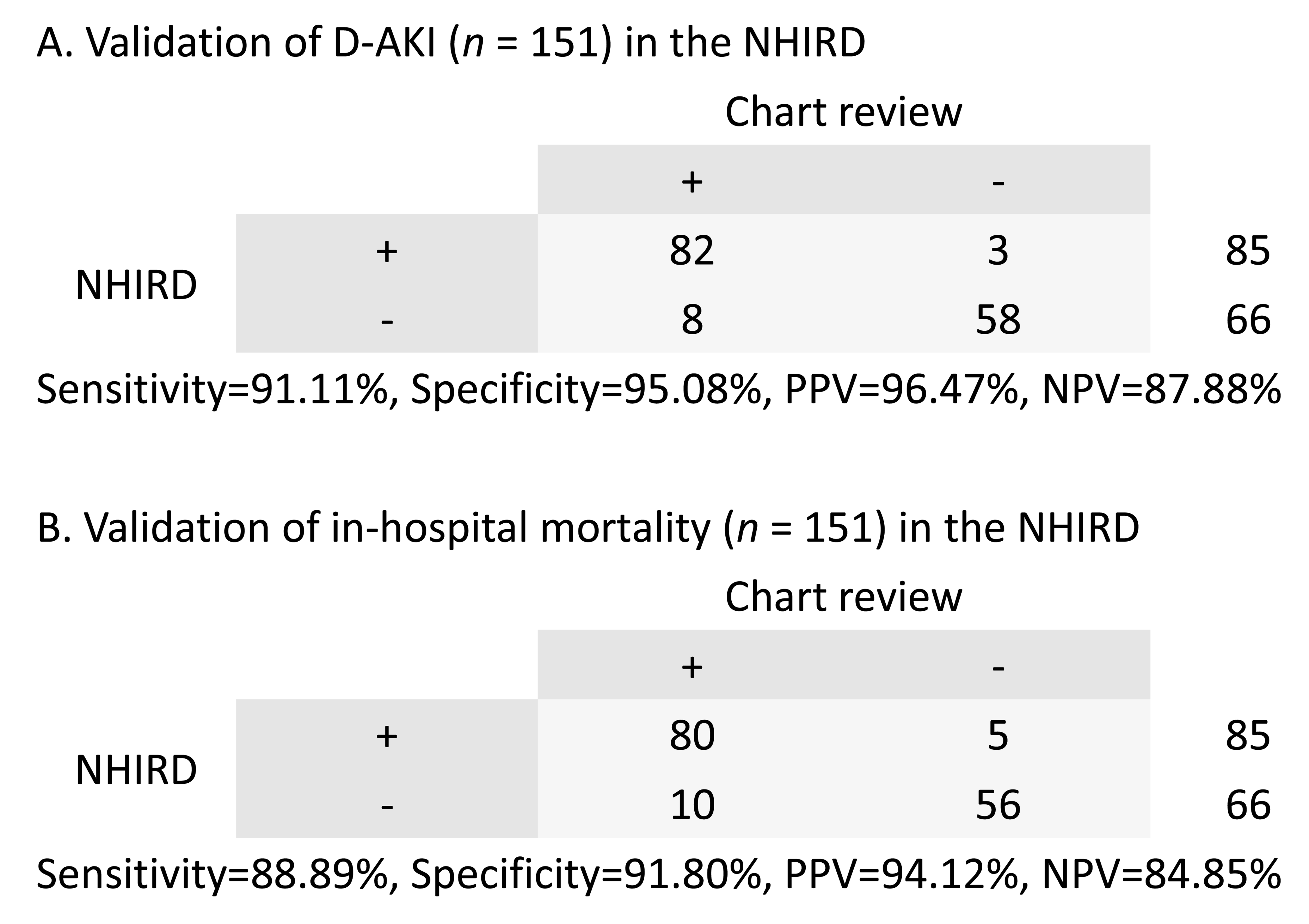

Supplement: S2 Fig — Validation of D-AKI (A) and in-hospital mortality (B). (TIF) [file pone.0212352.s002.tif]

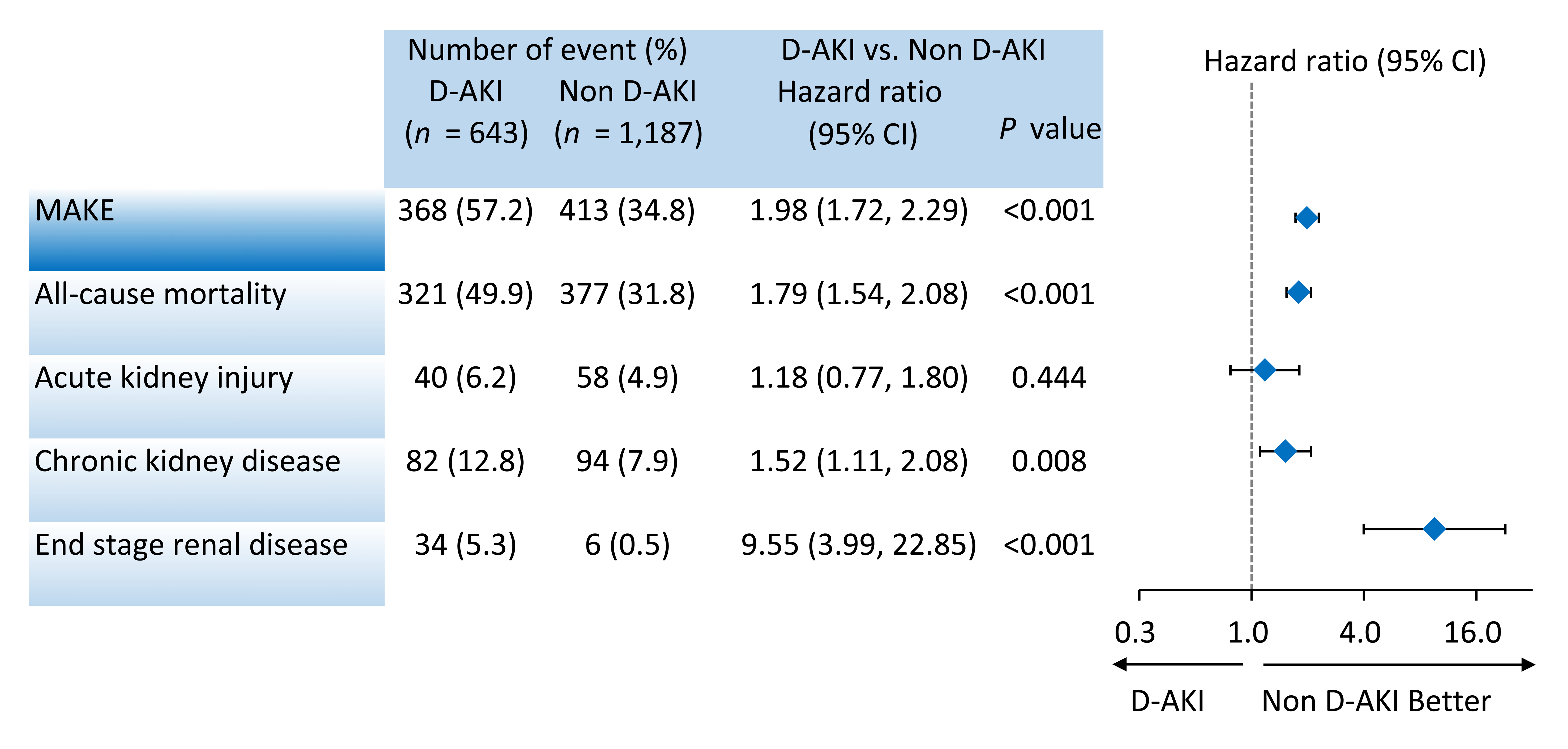

Supplement: S3 Fig — (TIF) [file pone.0212352.s003.tif]

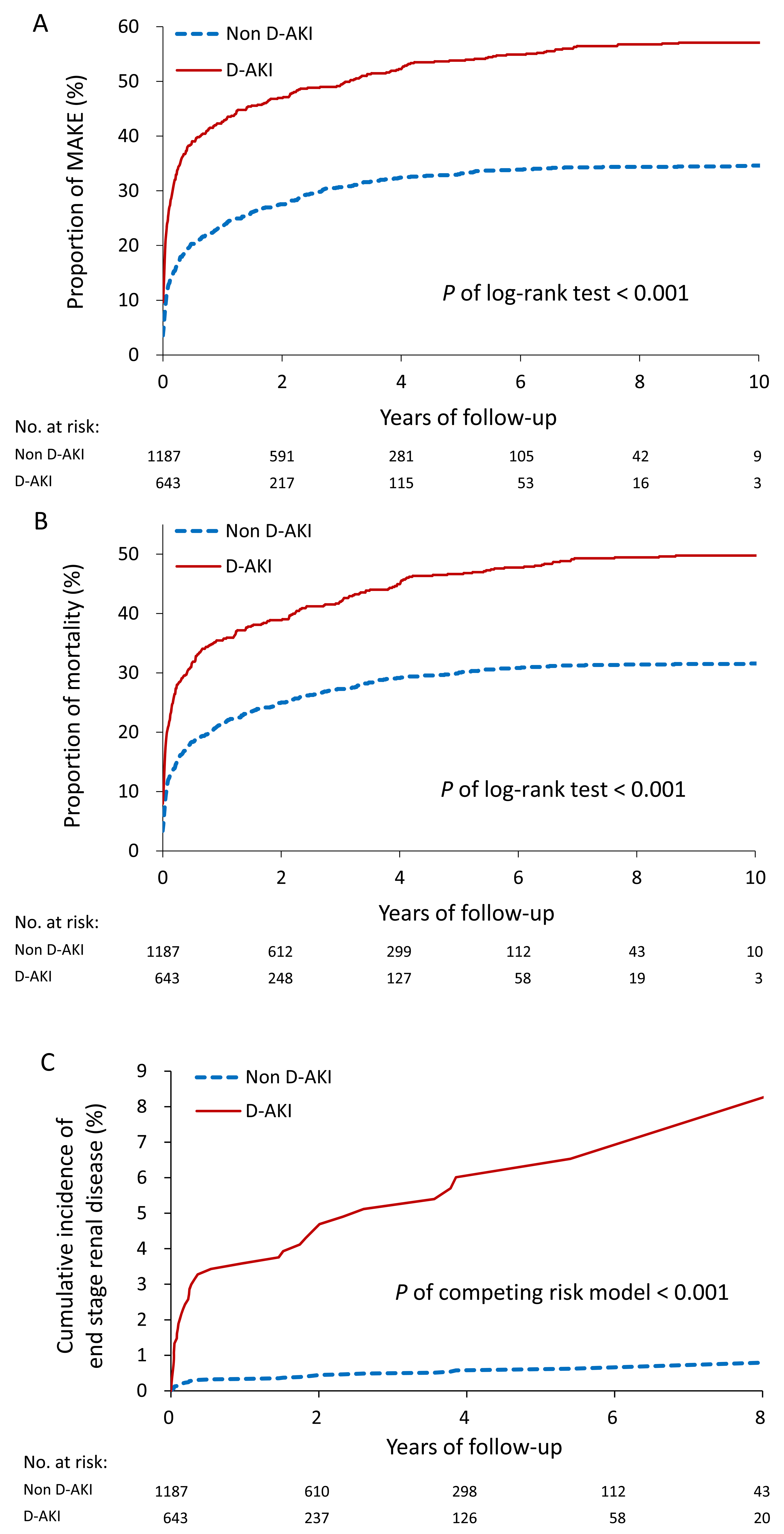

Supplement: S4 Fig — Proportion of major adverse kidney events (A) and all-cause mortality (B), cumulative incidence of end stage renal disease (C) during follow-up in the D-AKI and non D-AKI patients of patients with all indications of ECMO. (JPG) [file pone.0212352.s004.jpg]

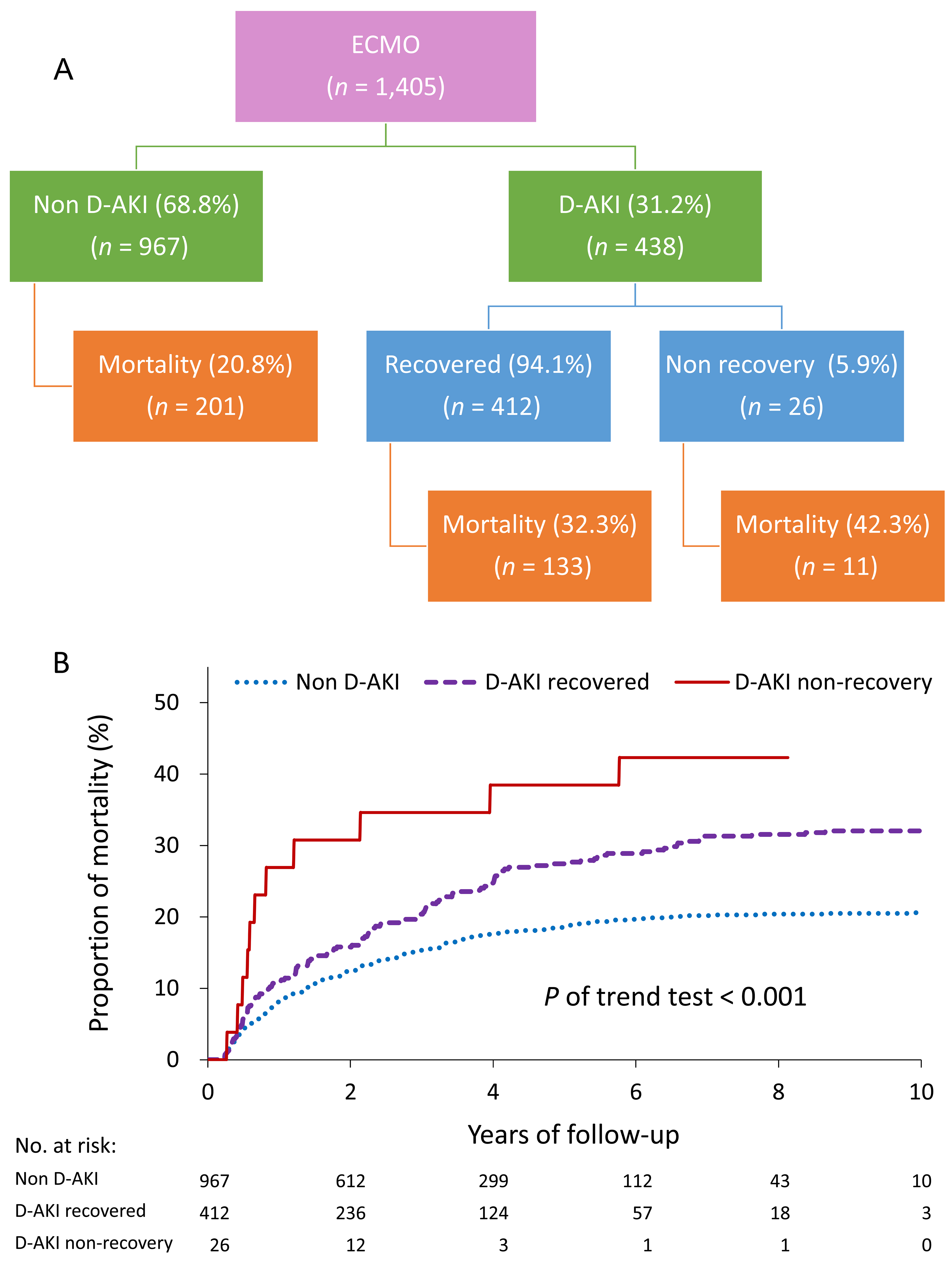

Supplement: S5 Fig — The mortality rate was shown by the stratification of renal function (A) along with a log-rank trend test (B) of patients with all indications of ECMO. (JPG) [file pone.0212352.s005.jpg]
