# Supplementary material for: Long-term outcomes after extracorporeal membrane oxygenation in patients with dialysis-requiring acute kidney injury: A cohort study
Source: PLoS One. 2019 Mar 13;14(3):e0212352. doi: 10.1371/journal.pone.0212352 (PMC6415889; doi:10.1371/journal.pone.0212352)
Supplement: S1 Table — (DOCX) [file pone.0212352.s006.docx]

**S1 Table.** ICD-9-CM code used for diagnosis in the current study

| **Variable** | **ICD-9-CM code** |
| --- | --- |
| Acute kidney injury | 584.xx |
| Chronic kidney disease | 580.xx–589.xx, 403.xx–404.xx, 016.0x, 095.4x, 236.9x, 250.4x, 274.1x, 442.1x, 447.3x, 440.1x, 572.4x, 642.1x, 646.2x, 753.1x, 283.11, 403.01, 404.02, 446.21 |
| End stage renal disease | 585.xx (Catastrophic illness card) |
| Cardiogenic shock | 411.0, 411.1, 411.8, 411.81, 411.89, 412, 413.0, 413.1, 413.9, 414.0, 414.00, 414.01, 414.06, 414.2, 414.3, 414.4, 414.8, 414.9, V458.1, V458.2, 415.0, 415.1, 415.12, 415.13, 415.19, 416.0, 416.1, 416.2, 416.8, 416.9, 417.0, 417.1, 417.8, 417.9, V125.5, 427.0, 427.1, 427.2, 427.31, 427.32, 427.60, 427.61, 427.69, 427.81, 427.89, 427.9, 785.0, 785.1, 427.41, 427.42, 427.5, 398.91, 428.0, 428.1, 428.20, 428.21, 428.22, 428.23, 428.30, 428.31, 428.32, 428.33, 428.40, 428.41, 428.42, 428.43 and 428.9 |
| Myocarditis | 328.2, 364.0, 364.1, 364.2, 364.3, 742.0, 742.1, 742.2, 742.3, 112.81, 115.03, 115.04, 115.13, 115.14, 115.93, 115.94, 130.3, 391.0, 391.1, 391.2, 391.8, 391.9, 392.0, 393, 398.0, 398.90, 398.99, 420.0, 420.90, 420.91, 420.99, 421.0, 421.1, 421.9, 422.0, 422.90, 422.91, 422.92, 422.93, 422.99, 423.0, 423.1, 423.2, 423.3, 423.8, 423.9, 425.0, 425.1, 425.11, 425.18, 425.2, 425.3, 425.4, 425.7, 425.8, 425.9 and 429.0 |
| Acute myocardial infarction | 410.0, 410.00, 410.01, 410.02, 410.1, 410.10, 410.11, 410.12, 410.2, 410.20, 410.21, 410.22, 410.3, 410.30, 410.31, 410.32, 410.4, 410.40, 410.41, 410.42, 410.5, 410.50, 410.51, 410.52, 410.6, 410.60, 410.61, 410.62, 410.7, 410.70, 410.71, 410.72, 410.8, 410.80, 410.81, 410.82, 410.9, 410.90, 410.91 and 410.92 |
| Respiratory | 020.0, 003.22, 020.3, 020.4, 020.5, 020.8, 020.9, 021.2, 021.8, 021.9, 022.1, 022.8, 022.9, 023.0, 023.1, 023.2, 023.3, 023.8, 023.9, 024, 025, 026.0, 026.9, 027.0, 027.1, 027.2, 027.8, 027.9, 030.0, 030.1, 030.2, 030.3, 030.8, 030.9, 031.0, 031.2, 031.8, 031.9, 032.0, 032.1, 032.2, 032.3, 032.89, 032.9, 033.0, 033.1, 033.8, 033.9, 034.0, 034.1, 036.3, 036.81, 036.89, 036.9, 037, 039.1, 039.2, 039.3, 039.4, 039.8, 039.9, 040.0, 040.1, 040.2, 040.3, 040.42, 040.81, 040.82, 040.89, 041.0, 041.0, 041.1, 041.2, 041.3, 041.4, 041.5, 041.9, 041.1, 041.10, 041.11, 041.12, 041.19, 041.2, 041.3, 041.4, 041.41, 041.42, 041.43, 041.49, 041.5, 041.6, 041.7, 041.8, 041.81, 041.82, 041.83, 041.84, 041.85, 041.86, 041.89, 041.9, 052.1, 055.1, 073.0, 083.0, 112.4, 114.0, 114.4, 114.5, 115.5, 115.15, 115.95, 130.4, 136.3, 390, 392.9, 460, 461.0, 461.1, 461.2, 461.3, 461.8, 461.9, 462, 464.0, 464.0, 464.1, 464.10, 464.11, 464.20, 464.21, 464.30, 464.31, 464.4, 464.50, 464.51, 465.0, 465.8, 465.9, 473.0, 473.1, 473.2, 473.3, 473.8, 473.9, 480.0, 480.1, 480.2, 480.3, 480.8, 480.9, 481, 482.0, 482.1, 482.2, 482.3, 482.30, 482.31, 482.32, 482.39, 482.4, 482.40, 482.41, 482.42, 482.49, 482.8, 482.81, 482.82, 482.83, 482.84, 482.89, 482.9, 483, 483.0, 483.1, 483.8, 484.1, 484.3, 484.5, 484.6, 484.7, 484.8, 485, 486, 487.0, 487.1, 487.8, 488, 488.0, 488.1, 488.2, 488.9, 488.1, 488.11, 488.12, 488.19, 488.81, 488.82, 488.89, 495.0, 495.1, 495.2, 495.3, 495.4, 495.5, 495.6, 495.7, 495.8, 495.9, 500, 501, 502, 503, 504, 505, 506.0, 506.1, 506.2, 506.3, 506.4, 506.9, 507.1, 507.8, 508.0, 508.1, 508.2, 508.8, 508.9, 510.0, 510.9, 511.0, 511.1, 511.8, 511.89, 511.9, 512.0, 512.8, 512.81, 512.82, 512.83, 512.84, 512.89, 513.0, 517.1, 517.3, 518.0, 518.1, 518.2, 518.5, 518.51, 518.52, 518.53, 518.81, 518.82, 518.83, 518.84, 784.91, 795.3, 795.31, 795.39, 799.1, V090, V091, V092, V093, V094, V095.0, V095.1, V096, V097.0, V097.1, V098.0, V098.1, V099.0, V099.1, V120.4, V461, V461.1, V461.2, V461.3, V461.4 and V462 |
| Trauma | 800.xx–994.xx |
| Diabetes mellitus | 250.xx |
| Hypertension | 401.xx–405.xx |
| Heart failure | 428.xx |
| Coronary artery disease | 410.xx–414.xx |
| Prior myocardial infarction | 410.xx, 412.xx |
| Atrial fibrillation | 427.31 |
| Peripheral arterial disease | 440.0x, 440.2x, 440.3x, 440.8x, 440.9x, 443.xx, 444.0x, 444.22, 444.8x, 447.8x, 447.9x |
| Stroke | 430.xx–437.xx |
| Ischemic stroke | 433.xx–437.xx |
| Hemorrhage stroke | 430.xx–432.xx |
| Coagulopathy | 286.0, 286.1, 286.2, 286.3 (Catastrophic illness card) |
| Chronic obstructive pulmonary disease | 491.xx, 492.xx, 496.xx |
| Liver cirrhosis | 571.2x, 571.5x, 571.6x |
| Malignancy | 140.xx–208.xx (Catastrophic illness card) |
| Sepsis | 038.xx, 790.7 |
| Respiratory failure | 518.xx |
