# Supplementary material for: Long-term outcomes after extracorporeal membrane oxygenation in patients with dialysis-requiring acute kidney injury: A cohort study
Source: PLoS One. 2019 Mar 13;14(3):e0212352. doi: 10.1371/journal.pone.0212352 (PMC6415889; doi:10.1371/journal.pone.0212352)
Supplement: S2 Table — (DOCX) [file pone.0212352.s007.docx]

**S2 Table. Demographic and clinical characteristics of patients with all indications of ECMO**

| Variable | | | Total  (*n* = 4,516) | D-AKI  (*n* = 2,432) | Non D-AKI  (*n* = 2,084) | | *P* |
| --- | --- | --- | --- | --- | --- | --- | --- |
| **Age (years)** | | | 55.6±16.2 | 56.7±16.0 | 54.4±16.2 | | <0.001 |
| **Age group** | | |  |  |  | | <0.001 |
|  | | **≤ 40 yrs.** | 851 (18.8) | 409 (16.8) | 442 (21.2) |  | |
|  | | **41–50 yrs.** | 721 (16.0) | 384 (15.8) | 337 (16.2) | |  |
|  | | **51–60 yrs.** | 1,089 (24.1) | 563 (23.1) | 526 (25.2) | |  |
|  | | **61–70 yrs.** | 877 (19.4) | 502 (20.6) | 375 (18.0) | |  |
|  | | **71–80 yrs.** | 713 (15.8) | 424 (17.4) | 289 (13.9) | |  |
|  | | **> 80 yrs.** | 265 (5.9) | 150 (6.2) | 115 (5.5) | |  |
| **Gender** | | |  |  |  | | 0.567 |
|  | | **Male** | 3,159 (70.0) | 1,710 (70.3) | 1,449 (69.5) | |  |
|  | | **Female** | 1,357 (30.0) | 722 (29.7) | 635 (30.5) | |  |
| **ECMO indication** | | |  |  |  | | 0.001 |
|  | | **CV (Cardiogenic shock,**  **myocarditis or AMI)** | 1,209 (26.8) | 597 (24.5) | 612 (29.4) | |  |
|  | | **Post-cardiotomy shock** | 2,042 (45.2) | 1,162 (47.8) | 880 (42.2) | |  |
|  | | **Respiratory** | 946 (20.9) | 509 (20.9) | 437 (21.0) | |  |
|  | | **Trauma** | 199 (4.4) | 105 (4.3) | 94 (4.5) | |  |
|  | | **Others** | 120 (2.7) | 59 (2.4) | 61 (2.9) | |  |
| **Comorbid conditions** | | |  |  |  | |  |
|  | | **Diabetes mellitus** | 1,132 (25.1) | 637 (26.2) | 495 (23.8) | 0.059 | |
|  | | **Hypertension** | 1,514 (33.5) | 827 (34.0) | 687 (33.0) | | 0.461 |
|  | | **Heart failure** | 733 (16.2) | 439 (18.1) | 294 (14.1) | | <0.001 |
|  | | **Coronary artery disease** | 2,212 (49.0) | 1,210 (49.8) | 1,002 (48.1) | | 0.262 |
|  | | **Prior myocardial infarction** | 468 (10.4) | 283 (11.6) | 185 (8.9) | | 0.002 |
|  | | **Atrial fibrillation** | 408 (9.0) | 230 (9.5) | 178 (8.5) | | 0.284 |
|  | | **Peripheral arterial disease** | 135 (3.0) | 80 (3.3) | 55 (2.6) | | 0.201 |
|  | | **Prior stroke** | 397 (8.8) | 232 (9.5) | 165 (7.9) | | 0.055 |
|  | | **Coagulopathy** | 192 (4.3) | 119 (4.9) | 73 (3.5) | | 0.021 |
|  | | **Chronic obstructive**  **pulmonary disease** | 267 (5.9) | 155 (6.4) | 112 (5.4) | | 0.156 |
|  | | **Liver cirrhosis** | 136 (3.0) | 96 (3.9) | 40 (1.9) | | <0.001 |
|  | | **Malignancy** | 283 (6.3) | 149 (6.1) | 134 (6.4) | | 0.675 |
|  | | **Charlson’s score** | 2.1±2.0 | 2.3±2.0 | 1.9±1.9 | | <0.001 |
| **Study year** | | |  |  |  | | 0.681 |
|  | **2003–2006** | | 498 (11.0) | 264 (10.9) | 234 (11.2) |  | |
|  | **2007–2010** | | 1,878 (41.6) | 1,001 (41.2) | 877 (42.1) | |  |
|  | **2011–2013** | | 2,140 (47.4) | 1,167 (48.0) | 973 (46.7) | |  |
| **Hospital level** | | |  |  |  | | 0.001 |
|  | **Medical center** | | 3,229 (71.5) | 1,791 (73.6) | 1,438 (69.0) |  | |
|  | **District / regional hospital** | | 1,287 (28.5) | 641 (26.4) | 646 (31.0) | |  |
| **Follow-up years** | | | 1.0±1.9 | 0.5±1.5 | 1.5±2.2 | | <0.001 |

D-AKI, dialysis-dependent acute kidney injury; ECMO, extracorporeal membrane oxygenation; CV, cardiovascular; AMI, acute myocardial infarction.
