# Supplementary material for: Long-term outcomes after extracorporeal membrane oxygenation in patients with dialysis-requiring acute kidney injury: A cohort study
Source: PLoS One. 2019 Mar 13;14(3):e0212352. doi: 10.1371/journal.pone.0212352 (PMC6415889; doi:10.1371/journal.pone.0212352)
Supplement: S3 Table — (DOCX) [file pone.0212352.s008.docx]

**S3 Table. In-hospital outcomes of patients with all indications of ECMO**

| **Outcome** | | **Total**  **(*n* = 4,516)** | **D-AKI**  **(*n* = 2,432)** | **Non D-AKI**  **(*n* = 2,084)** | **D-AKI *vs*. Non D-AKI** |  |
| --- | --- | --- | --- | --- | --- | --- |
|  |  |  |  |  | ***B* / OR (95% CI)^1^** | ***P* value** |
| **Categorical parameter** | |  |  |  |  |  |
|  | **In-hospital mortality** | 2,686 (59.5) | 1,789 (73.6) | 897 (43.0) | 3.67 (3.22–4.18) | <0.001 |
|  | **New onset ischemic**  **stroke** | 177 (3.9) | 89 (3.7) | 88 (4.2) | 0.78 (0.57–1.07) | 0.117 |
|  | **New onset**  **hemorrhagic stroke** | 106 (2.3) | 48 (2.0) | 58 (2.8) | 0.68 (0.46–1.01) | 0.054 |
|  | **Sepsis** | 1,023 (22.7) | 648 (26.6) | 375 (18.0) | 1.81 (1.55–2.11) | <0.001 |
|  | **Fasciotomy or**  **amputation** | 80 (1.8) | 59 (2.4) | 21 (1.0) | 2.36 (1.42–3.94) | 0.001 |
|  | **Respiratory failure** | 753 (16.7) | 435 (17.9) | 318 (15.3) | 1.15 (0.98–1.35) | 0.095 |
|  | **IABP** | 1,978 (43.8) | 1,093 (44.9) | 885 (42.5) | 1.18 (1.02–1.37) | 0.031 |
|  | **Massive blood**  **transfusion, PRBC >**  **10 Units** | 2,638 (58.4) | 1,679 (69.0) | 959 (46.0) | 2.73 (2.38–3.12) | <0.001 |
| **Continuous parameter** | |  |  |  |  |  |
|  | **PRBC amount** | 18.2±17.3 | 22.0±19.1 | 13.8±13.8 | 7.21 (6.28, 8.13) | <0.001 |
|  | **FFP amount** | 13.7±20.5 | 17.8±23.7 | 8.9±14.6 | 7.81 (6.68, 8.93) | <0.001 |
|  | **Platelet amount** | 12.3±21.0 | 15.6±23.9 | 8.5±16.3 | 6.85 (5.68, 8.03) | <0.001 |
|  | **ECMO support**  **duration (days)** | 5.5±3.3 | 5.8±3.4 | 5.1±3.0 | 0.55 (0.36, 0.74) | <0.001 |
|  | **Ventilator (days)** | 15.7±16.6 | 16.9±18.1 | 14.3±14.6 | 2.04 (1.09, 3.00) | <0.001 |
|  | **ICU duration (days)** | 17.1±16.9 | 18.0±18.2 | 16.1±15.3 | 1.33 (0.36, 2.30) | 0.007 |
|  | **Hospital stay (days)** | 27.5±31.0 | 26.9±32.1 | 28.2±29.6 | -2.49 (-4.26, -0.72) | 0.006 |
|  | **Inpatient medical**  **expenditure**  **(NTD×10^4^)** | 94.5±70.2 | 104.9±76.4 | 82.5±60.0 | 18.44 (14.62, 22.27) | <0.001 |

^1^ Adjusted for variables listed in Table 1 except follow up years;

D-AKI, dialysis-dependent acute kidney injury; *B,* regression coefficient; OR, odds ratio; CI, confidence interval; IABP, intra-aortic balloon pump; PRBC, packed red blood cells; FFP, fresh frozen plasma; ICU, intensive care unit; NTD, New Taiwan Dollar.
