# Supplementary material for: Long-term outcomes after extracorporeal membrane oxygenation in patients with dialysis-requiring acute kidney injury: A cohort study
Source: PLoS One. 2019 Mar 13;14(3):e0212352. doi: 10.1371/journal.pone.0212352 (PMC6415889; doi:10.1371/journal.pone.0212352)
Supplement: S4 Table — (DOCX) [file pone.0212352.s009.docx]

**S4 Table. Risk factor analysis of D-AKI of patients with all indications of ECMO**

| **Variable** | **Odds ratio** | **95% CI** | ***P* value** |
| --- | --- | --- | --- |
| **Age (per 10 years)** | 1.095 | 1.055–1.137 | <0.001 |
| **Indication (Ref: CV)** |  |  |  |
| **Post-cardiotomy shock** | 1.277 | 1.105–1.476 | <0.001 |
| **Respiratory** | 1.222 | 1.027–1.455 | 0.024 |
| **Trauma** | 1.330 | 0.979–1.806 | 0.068 |
| **Others** | 0.990 | 0.676–1.449 | 0.958 |
| **Heart failure** | 1.276 | 1.081–1.506 | 0.004 |
| **Liver cirrhosis** | 2.098 | 1.435–3.067 | <0.001 |
| **Medical center** | 1.241 | 1.089–1.415 | 0.001 |

D-AKI, dialysis-dependent acute kidney injury; CI, confidence interval; CV, cardiovascular.
